# Supplementary material for: Longitudinal SARS-CoV-2 antibody study using the Easy Check COVID-19 IgM/IgG™ lateral flow assay
Source: PLoS One. 2021 Mar 4;16(3):e0247797. doi: 10.1371/journal.pone.0247797 (PMC7932143; doi:10.1371/journal.pone.0247797)
Supplement: Attachment — Submitted filename: Response to Reviewers.pdf [file pone.0247797.s002.pdf]

February 8th, 2021

Dear Editors,

Thank you for your feedback regarding our manuscript, Longitudinal SARS-CoV-2 Antibody Study Using the Easy Check COVID-19 IgM/IgG Lateral Flow Assay. You have made very good points regarding several aspects of our paper. We will address each point below in blue.

1. Please ensure that your manuscript meets PLOS ONE's style requirements, including those for file naming. The PLOS ONE style templates can be found at

Author Response: We have revised our manuscript to ensure that it meets PLOS ONE's style requirements, including those for file naming.

2. Please include the date range on which samples were originally collected as well as the date(s) you obtained the retrospective data.

Author Response: We have added the date range on which samples were originally collected as well as the dates we obtained the retrospective data.

3. Thank you for stating the following in the Acknowledgments Section of your manuscript:

"This research was supported in part by the San Diego Center for AIDS Research (SD CFAR) Translational Virology Core, an NIH-funded program (P30 AI036214)."

We note that you have provided funding information that is not currently declared in your Funding Statement. However, funding information should not appear in the Acknowledgments section or other areas of your manuscript. We will only publish funding information present in the Funding Statement section of the online submission form.

Please remove any funding-related text from the manuscript and let us know how you would like to update your Funding Statement. Currently, your Funding Statement reads as follows:

"The authors received no specific funding for this work."

Please include your amended statements within your cover letter; we will change the online submission form on your behalf.

Author Response: We apologize for the confusion. One of our authors, Stephen Rawlings salary is covered by the SD CFAR Tranlsational Virology Core, an NIH-funded program. This is not specific funding for our project but rather a general funding source such that Stephen can execute research related to SD CFAR Translational Virology Core initiatives. Our funding statement remains that we received no specific funding for this work. We will remove funding-related text from the manuscript.

4. Thank you for stating the following in the Competing Interests section:

"I have read the journal's policy and the authors of this manuscript have the following competing interests: Renee Higgins, Florence Lee and Dena Marrinucci are employees of Truvian Sciences, the company that sells Easy Check. Kiang-Teck J. Yeo is a member of the Scientific Advisory Board of Truvian and has received honoraria and equities from Truvian."

Please confirm that this does not alter your adherence to all PLOS ONE policies on sharing data and materials, by including the following statement: "This does not alter our adherence to PLOS ONE policies on sharing data and materials." (as detailed online in our guide for authors http://journals.plos.org/plosone/s/competing-interests). If there are restrictions on sharing of data and/or materials, please state these. Please note that we cannot proceed with consideration of your article until this information has been declared.

Please include your updated Competing Interests statement in your cover letter; we will change the online submission form on your behalf.

Please know it is PLOS ONE policy for corresponding authors to declare, on behalf of all authors, all potential competing interests for the purposes of transparency. PLOS defines a competing interest as anything that interferes with, or could reasonably be perceived as interfering with, the full and objective presentation, peer review, editorial decision-making, or publication of research or non-research articles submitted to one of the journals. Competing interests can be financial or non-financial, professional, or personal. Competing interests can arise in relationship to an organization or another person. Please follow this link to our website for more details on competing interests: http://journals.plos.org/plosone/s/competing-interests

Author Response: Updated Competing Interests statement is as follows: I have read the journal's policy and the authors of this manuscript have the following competing interests: Renee Higgins, Florence Lee and Dena Marrinucci are employees of Truvian Sciences, the company that sells Easy Check. Kiang-Teck J. Yeo is a member of the Scientific Advisory Board of Truvian and has received honoraria and equities from Truvian. This does not alter our adherence to PLOS ONE policies on sharing data and materials.

5. We note that Figures 1 and 2 in your submission contain copyrighted images. All PLOS content is published under the Creative Commons Attribution License (CC BY 4.0), which means that the manuscript, images, and Supporting Information files will be freely available online, and any third party is permitted to access, download, copy, distribute, and use these materials in any way, even commercially, with proper attribution. For more information, see our copyright guidelines: http://journals.plos.org/plosone/s/licenses-and-copyright.

We require you to either (1) present written permission from the copyright holder to publish these figures specifically under the CC BY 4.0 license, or (2) remove the figures from your submission:

(1) You may seek permission from the original copyright holder of Figures 1 and 2 to publish the content specifically under the CC BY 4.0 license.

We recommend that you contact the original copyright holder with the Content Permission Form (http://journals.plos.org/plosone/s/file?id=7c09/content-permission-form.pdf) and the following text:

"I request permission for the open-access journal PLOS ONE to publish XXX under the Creative Commons Attribution License (CCAL) CC BY 4.0 (http://creativecommons.org/licenses/by/4.0/). Please be aware that this license allows unrestricted use and distribution, even commercially, by third parties. Please reply and provide explicit written permission to publish XXX under a CC BY license and complete the attached form."

Please upload the completed Content Permission Form or other proof of granted permissions as an "Other" file with your submission.

In the figure caption of the copyrighted figure, please include the following text: "Reprinted from [ref] under a CC BY license, with permission from [name of publisher], original copyright [original copyright year]."

(2) If you are unable to obtain permission from the original copyright holder to publish these figures under the CC BY 4.0 license or if the copyright holder's requirements are incompatible with the CC BY 4.0 license, please either i) remove the figure or ii) supply a replacement figure that complies with the CC BY 4.0 license. Please check copyright information on all replacement figures and update the figure caption with source information. If applicable, please specify in the figure caption text when a figure is similar but not identical to the original image and is therefore for illustrative purposes only.

Author Response: We have received permission for PLOS One to publish Figures 1 and 2 from AJCP. We have uploaded proof of granted permissions as an "Other" file with our submission.

6. We note that you have indicated that data from this study are available upon request. PLOS only allows data to be available upon request if there are legal or ethical restrictions on sharing data publicly. For information on unacceptable data access restrictions, please see http://journals.plos.org/plosone/s/data-availability#loc-unacceptable-data-access-restrictions.

In your revised cover letter, please address the following prompts:

a) If there are ethical or legal restrictions on sharing a de-identified data set, please explain them in detail (e.g., data contain potentially identifying or sensitive patient information) and who has imposed them (e.g., an ethics committee). Please also provide contact information for a data access committee, ethics committee, or other institutional body to which data requests may be sent.

b) If there are no restrictions, please upload the minimal anonymized data set necessary to replicate your study findings as either Supporting Information files or to a stable, public repository and provide us with the relevant URLs, DOIs, or accession numbers. Please see http://www.bmj.com/content/340/bmj.c181.long for guidelines on how to de-identify and prepare

clinical data for publication. For a list of acceptable repositories, please see http://journals.plos.org/plosone/s/data-availability#loc-recommended-repositories.

We will update your Data Availability statement on your behalf to reflect the information you provide.

Author Response: There are no legal or ethical restrictions on sharing our data publicly. We have uploaded our minimal anonymized data set as supporting information files.

Reviewer #1: the paper appears to add some valuable new information regarding a rapid test for antibody positivity to SARS-CoV2 as well as some information about duration of IgM and IgG antibodies. In general it is well written. there are some critiques as follows:

1. The assay is not compared to another gold standard assay within this study. It was validated in another study but the authors should at least discuss the potential for false positives and false negatives within their data set based on prior validation

   Author Response: Thank you for your feedback. This is a valid point. We have added a statement to our manuscript to highlight the possibility of false positives and false negatives within our data set.

2. The gold standard used to determine positivity for COVID 19 should be provided

   Author Response: There were multiple gold standard PCR tests used across the 3 sites. We have updated our methods section to include a list of all PCR tests used.

3. There is little data on the clinical course of the patients - was any data available?

   Author Response: Complete data on subject symptom severity were not available for all samples. However, within cohorts that had symptom severity, the majority of participants had mild and moderate symptoms, and very few had severe symptoms—similar to the distribution of clinical outcomes for the pandemic as a whole. We do not have complete follow-up data on all participants either but are only aware of 17 fatalities.

4. The authors should discuss what their findings add to the existing literature - Conclusions are drawn about the duration of antibody positivity but the number of patients followed for over 90 days is limited

   Author Response: Thank you for your feedback. We have added text to our manuscript to highlight what our findings add to the existing literature as well as discussing the limitations of the findings.

5. The final figure is very hard to interpret

   Author Response: We have updated figure 5 to make the message clearer. We're open to additional feedback if it is still difficult to follow.

We look forward to hearing your decision on our manuscript. Thank you for your time reviewing our submission and response.

Kind regards,

Reneé Higgins, PhD
Manager, Scientific Affairs
Truvian
10300 Campus Point Dr
Suite 190
San Diego, CA 92121
